# Effects of iron loading on muscle: genome-wide mRNA expression profiling in the mouse

**DOI:** 10.1186/1471-2164-8-379

**Published:** 2007-10-19

**Authors:** Alejandra Rodriguez, Mika Hilvo, Leena Kytömäki, Robert E Fleming, Robert S Britton, Bruce R Bacon, Seppo Parkkila

**Affiliations:** 1 Institute of Medical Technology, University of Tampere and Tampere University Hospital, Tampere, Finland; 2 Turku Centre for Biotechnology, University of Turku, Turku, Finland; 3 Department of Pediatrics, Saint Louis University School of Medicine, St. Louis, USA; 4 Edward A. Doisy Department of Biochemistry and Molecular Biology, Saint Louis University School of Medicine, St. Louis, USA; 5 Department of Internal Medicine, Saint Louis University School of Medicine, St. Louis, USA; 6 Department of Clinical Chemistry, University of Oulu, Oulu, Finland

## Abstract

**Background:**

Hereditary hemochromatosis (HH) encompasses genetic disorders of iron overload characterized by deficient expression or function of the iron-regulatory hormone hepcidin. Mutations in 5 genes have been linked to this disease: *HFE*, *TFR2 *(encoding transferrin receptor 2), *HAMP *(encoding hepcidin), *SLC40A1 *(encoding ferroportin) and *HJV *(encoding hemojuvelin). Hepcidin inhibits iron export from cells into plasma. Hemojuvelin, an upstream regulator of hepcidin expression, is expressed in mice mainly in the heart and skeletal muscle. It has been suggested that soluble hemojuvelin shed by the muscle might reach the liver to influence hepcidin expression. Heart muscle is one of the target tissues affected by iron overload, with resultant cardiomyopathy in some HH patients. Therefore, we investigated the effect of iron overload on gene expression in skeletal muscle and heart using Illumina™ arrays containing over 47,000 probes. The most apparent changes in gene expression were confirmed using real-time RT-PCR.

**Results:**

Genes with up-regulated expression after iron overload in both skeletal and heart muscle included angiopoietin-like 4, pyruvate dehydrogenase kinase 4 and calgranulin A and B. The expression of transferrin receptor, heat shock protein 1B and DnaJ homolog B1 were down-regulated by iron in both muscle types. Two potential hepcidin regulatory genes, hemojuvelin and neogenin, showed no clear change in expression after iron overload.

**Conclusion:**

Microarray analysis revealed iron-induced changes in the expression of several genes involved in the regulation of glucose and lipid metabolism, transcription and cellular stress responses. These may represent novel connections between iron overload and pathological manifestations of HH such as cardiomyopathy and diabetes.

## Background

It is crucial for the human body to maintain iron homeostasis. Since there is no adjustable mechanism to influence iron loss from the body, tight regulation of iron absorption at the intestinal level is vital [1]. In order to maintain iron balance, iron export from enterocytes, reticuloendothelial macrophages and hepatocytes into the blood stream has to be controlled as well. Functional derangement of proteins involved in these regulatory mechanisms can cause hereditary hemochromatosis (HH, OMIM-235200). This genetic disorder of iron overload is characterized by high transferrin saturation, low iron content in macrophages, and deposition of iron in several organs including the liver, heart, and pancreas. Causative mutations for HH have been described in several genes, namely *HFE*, *TFR2 *(encoding transferrin receptor 2), *HJV *(encoding hemojuvelin), and *HAMP *(encoding hepcidin) [2-7]. It has been proposed that these mutations cause deficient hepcidin synthesis [4,5,8,9].

The antimicrobial peptide hepcidin is the central regulator of iron metabolism. It is produced mainly in the liver and exerts its function by binding to the iron export protein, ferroportin, inducing its internalization and degradation [10]. Ferroportin is located in the cellular membranes of enterocytes, reticuloendothelial cells, hepatocytes and placental cells [11]. Therefore, hepcidin acts to decrease the export of iron from these cells into the circulation.

Hemojuvelin is a glycosyl phosphatidylinositol-anchored protein which belongs to the repulsive guidance molecule (RGM) protein family [4,12]. Recent studies suggest that hemojuvelin exists in two forms. One is a rarer full-length protein shed to the extracellular fluid, where it has a long half-life. The other is a smaller, membrane-associated disulfide-linked heterodimer, which is a more abundant but shorter-lived form composed of N- and C-terminal fragments [13,14]. According to latest studies the most common mutation in hemojuvelin (G320V) affects the targeting of the membrane-associated form and reduces the amount of the soluble form [15]. Interestingly, studies in cultured cells suggest that the two forms regulate hepcidin expression reciprocally by competing for a receptor binding site [14]. Evidence shows that hemojuvelin is a bone morphogenetic protein co-receptor, and its interaction with BMP initiates a signaling cascade that leads to regulation of hepcidin expression [16,17]. On the other hand, it has been observed that overexpressed hemojuvelin binds to the membrane receptor neogenin and that this interaction is required for the accumulation of iron in cultured cells [12]. Zhang *et al*. also showed that the G320V mutated hemojuvelin overexpressed *in vitro *was not able to bind neogenin, and that iron did not accumulate in the cells under these conditions. Furthermore, a recent study in cultured cells suggested that neogenin may mediate inhibition of hemojuvelin shedding in response to iron [18]. We have previously determined the sites of simultaneous expression of hemojuvelin and neogenin [19]. The highest expression of hemojuvelin transcript is found in the skeletal muscle and heart. Although *in vivo *evidence of a combined role of hemojuvelin and neogenin in iron homeostasis has not been provided yet, it has been suggested that hemojuvelin shed from skeletal muscle and heart by neogenin-dependent mechanism could reach the liver to influence hepcidin expression [12].

Cardiomyopathy develops in some HH patients [20]. In order to better understand the mechanisms behind pathological effects of iron overload in muscle cells, we have performed a genome-wide expression analysis of genes in skeletal muscle and heart of mice with or without dietary iron loading. Microarray data analysis identified several genes whose expression was either down- or up-regulated due to iron overload. These results may reveal novel links between iron overload and pathological manifestations of HH.

## Results

### Documentation of iron overload in the liver and heart of iron-fed mice

**Figure 1:**
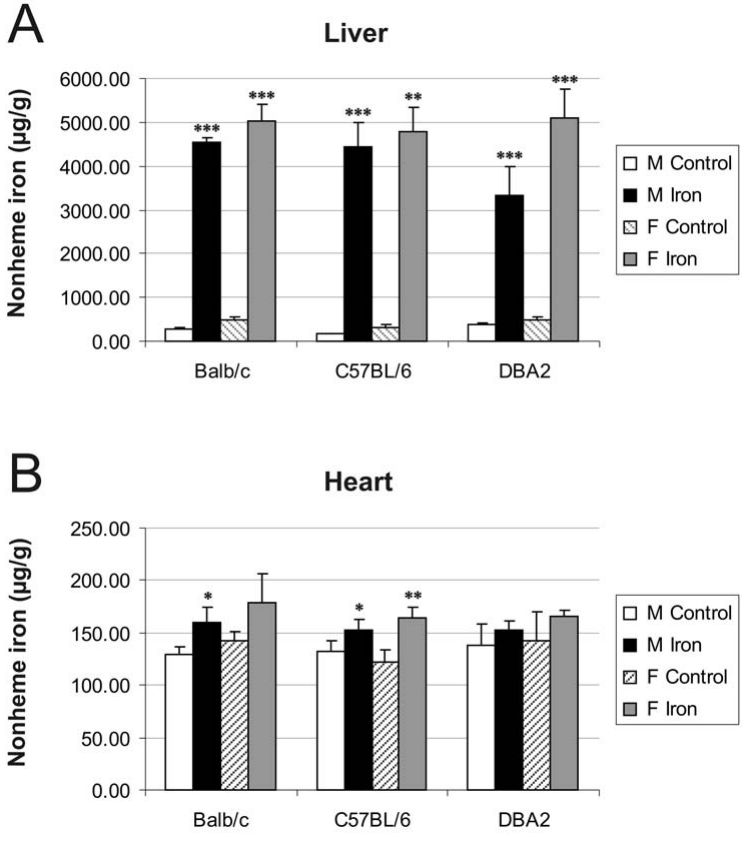
**Hepatic and cardiac non-heme iron concentrations**. Iron contents were studied in three strains of male and female mice fed either the control or high-iron diet. The result values are expressed as mean +/- standard deviation. Statistical significant differences relative to control diet fed mice were determined. *p < 0,05; **p < 0,01; ***p < 0,001. F = female; M = male.

The mice were fed either standard (0.02% carbonyl iron) or high-iron (2% carbonyl iron) diet for 6 weeks. Iron concentrations of liver and heart specimens were determined to confirm the validity of the animal model. Figure 1A demonstrates that the livers of mice of all three strains were highly iron-loaded when fed an iron-rich diet. A much smaller increment in cardiac iron content after a high-iron diet was observed also in all three strains and in both genders (Figure 1B), although statistical significance was not reached in all the cases. Basal cardiac iron levels were lower than basal hepatic contents. In general, female mice showed slightly higher hepatic and cardiac iron levels than male mice.

### Identification and validation of changes in gene expression induced by dietary iron overload in skeletal muscle and heart

We obtained a list of 14 genes with iron-induced up-regulated expression in skeletal muscle (Table 1) and forty with down-regulated expression (Table 2). In the heart, iron loading resulted in the up-regulation of 35 genes (Table 3), while forty genes had down-regulated expression after iron overload (Table 4). There were seven genes which were up-regulated in both the heart and skeletal muscle, while nine genes were down-regulated in both tissues.

**Table 1:** Genes with up-regulated expression in skeletal muscle during iron overload

**Gene name**	**Symbol**	**Accession**.	**Fold change**
Calgranulin A, S100 calcium binding protein A8	S100a8	NM_013650	2.80
Calgranulin B, S100 calcium binding protein A9	S100a9	NM_009114	2.26
Stearoyl-Coenzyme A desaturase 1	Scd1	NM_009127	1.75
Adipsin, complement factor D	Adn	NM_013459	1.62
Myosin light polypeptide 2	Myl2	NM_010861	1.60
UDP-N-acetyl-alpha-D-galactosamine:polypeptide N-acetylgalactosaminyl transferase-like 2	Galntl2	XM_127638	1.56
cytochrome P450, family 26, subfamily b, polypeptide 1	Cyp26b1	NM_175475	1.49
cold inducible RNA binding protein	Cirbp	NM_007705	1.48
Cbp/p300-interacting transactivator, with Glu/Asp-rich carboxy-terminal domain, 2	Cited2	NM_010828	1.46
angiopoietin-like 4	Angptl4	NM_020581	1.45
epididymal protein Av381126		NM_183143	1.45
pyruvate dehydrogenase kinase, isoenzyme 4	Pdk4	NM_013743	1.40
myeloid/lymphoid or mixed lineage-leukemia translocation to 4 homolog (Drosophila)	Mllt4	XM_890447	1.40
6-phosphofructo-2-kinase/fructose-2,6-biphosphatase 3	Pfkfb3	NM_133232	1.40

Data obtained with 3 high-iron and 3 control samples.

**Table 2:** Genes with down-regulated expression in skeletal muscle during iron overload

**Gene name**	**Symbol**	**Accession**.	**Fold change**
major urinary protein 1	Mup1	NM_031188	-2.61
DnaJ (Hsp40) homolog, subfamily B, member 1	Dnajb1	NM_018808	-2.52
Heat shock protein 1B	Hspa1b	NM_010478	-2.40
solute carrier family 25 (mitochondrial carrier, phosphate carrier), member 25	Slc25a25	NM_146118	-2.22
major urinary protein 3	Mup3	NM_010845	-2.21
FBJ osteosarcoma oncogene	Fos	NM_010234	-2.10
heat shock protein 1, alpha	Hspca	NM_010480	-1.91
early growth response 3	Egr3	NM_018781	-1.79
metallothionein 1	Mt1	NM_013602	-1.78
heat shock protein 105	Hsp105	NM_013559	-1.72
RIKEN full-length enriched library, clone:A530098C11 product: hypothetical SAM (and some other nucleotide) binding motif containing protein		AK041301	-1.70
ERBB receptor feedback inhibitor 1	Errfi1	NM_133753	-1.69
inhibitor of DNA binding 1	Idb1	NM_010495	-1.66
Transthyretin	Ttr	NM_013697	-1.65
Kruppel-like factor 4	Klf4	NM_010637	-1.65
nuclear factor, interleukin 3, regulated	Nfil3	NM_017373	-1.64
cyclin-dependent kinase inhibitor 1A	Cdkn1a	NM_007669	-1.62
RIKEN full-length enriched library, clone:D830037I21 product:weakly similar to RING ZINC FINGER PROTEIN SMRZ [Homo sapiens]		AK052911	-1.61
protein phosphatase 1, regulatory subunit 10	Ppp1r10	NM_175934	-1.61
connective tissue growth factor	Ctgf	NM_010217	-1.59
serine (or cysteine) proteinase inhibitor, clade H, member 1	Serpinh1	NM_009825	-1.58
cerebellar degeneration-related 2	Cdr2	NM_007672	-1.58
neural precursor cell expressed, developmentally down-regulated gene 9	Nedd9	NM_017464	-1.58
apolipoprotein A-II	Apoa2	NM_013474	-1.54
DNA-damage-inducible transcript 4	Ddit4	NM_029083	-1.54
PDZ and LIM domain 1	Pdlim1	NM_016861	-1.51
activating transcription factor 3	Atf3	NM_007498	-1.49
heat shock protein 1A	Hspa1a	NM_010479	-1.48
heat shock protein 1	Hspb1	NM_013560	-1.48
neural precursor cell expressed, developmentally down-regulated gene 9	Nedd9	NM_017464	-1.47
actin, alpha, cardiac	Actc1	NM_009608	-1.46
inositol hexaphosphate kinase 3	Ihpk3	NM_173027	-1.45
kidney androgen regulated protein	Kap	NM_010594	-1.44
metallothionein 2	Mt2	NM_008630	-1.44
8430408G22Rik		NM_145980	-1.43
cyclin-dependent kinase inhibitor 1A	Cdkn1a	NM_007669	-1.42
G0/G1 switch gene 2	G0s2	NM_008059	-1.42
fos-like antigen 2	Fosl2	NM_008037	-1.42
procollagen, type I, alpha 1	Col1a1	NM_007742	-1.41
dysferlin interacting protein 1	Dysfip1	NM_026814	-1.40

Data obtained with 3 high-iron and 3 control samples.

**Table 3:** Genes with up-regulated expression in the heart during iron overload

**Gene name**	**Symbol**	**Accession**.	**Fold change**
myosin, light polypeptide 7, regulatory	Myl7	NM_022879	7.68 **
myosin, light polypeptide 4, alkali	Myl4	NM_010858	6.32 **
seminal vesicle secretion 5	Svs5	NM_009301	5.21 **
seminal vesicle protein 2	Svp2	NM_009300	4.25 **
myosin binding protein H-like	Mybphl	NM_026831	4.14 **
angiopoietin-like 4	Angptl4	NM_020581	2.79 *
seminal vesicle protein, secretion 2	Svs2	NM_017390	2.61 **
pyruvate dehydrogenase kinase, isoenzyme 4	Pdk4	NM_013743	2.06 *
S100 calcium binding protein A8 (calgranulin A)	S100a8	NM_013650	1.96 *
S100 calcium binding protein A9 (calgranulin B)	S100a9	NM_009114	1.95 *
3-hydroxy-3-methylglutaryl-Coenzyme A synthase 2	Hmgcs2	NM_008256	1.82 **
Ras-related associated with diabetes	Rrad	NM_019662	1.81 **
thioredoxin interacting protein	Txnip	NM_023719	1.78 *
secretory leukocyte protease inhibitor	Slpi	NM_011414	1.69 **
dickkopf homolog 3 (Xenopus laevis)	Dkk3	NM_015814	1.68 **
START domain containing 10	Stard10	NM_019990	1.68 **
D site albumin promoter binding protein	Dbp	NM_016974	1.65 *
lectin, galactose binding, soluble 4	Lgals4	NM_010706	1.65 **
cytochrome P450, family 26, subfamily b, polypeptide 1	Cyp26b1	NM_175475	1.62 **
2310043N10Rik		XM_979471	1.55 *
cold inducible RNA binding protein	Cirbp	NM_007705	1.49 *
FBJ osteosarcoma oncogene	Fos	NM_010234	1.49 **
2900060B14Rik			1.49 *
early growth response 1	Egr1	NM_007913	1.46 *
1810015C04Rik		NM_025459	1.45 *
seminal vesicle secretion 1	Svs1	NM_172888	1.44 **
Iroquois related homeobox 3 (Drosophila)	Irx3	NM_008393	1.43 **
	BC031353	NM_153584	1.43 *
folliculin interacting protein 1	Fnip1	NM_173753	1.42 **
myosin, heavy polypeptide 7, cardiac muscle, beta	Myh7	NM_080728	1.42 **
Cbp/p300-interacting transactivator, with Glu/Asp-rich carboxy-terminal domain, 2	Cited2	NM_010828	1.41*
2610035D17Rik		XM_990633	1.41 **
a disintegrin-like and metalloprotease (reprolysin type) with thrombospondin type 1 motif, 1	Adamts1	NM_009621	1.40 *
fructose bisphosphatase 2	Fbp2	NM_007994	1.40 *
2300009N04Rik			1.40 *

*Data obtained with 2 high-iron and 2 control samples.

** Data obtained with 3 high-iron and 3 control samples.

**Table 4:** Genes with down-regulated expression in the heart during iron overload

**Gene name**	**Symbol**	**Accession**.	**Fold change**
uncoupling protein 1, mitochondrial	Ucp1	NM_009463	-4.47 **
actin, alpha 1, skeletal muscle	Acta1	NM_009606	-2.79 *
chemokine (C-X-C motif) ligand 7	Cxcl7	NM_023785	-2.41 *
stearoyl-Coenzyme A desaturase 1	Scd1	NM_009127	-2.40 **
heat shock protein 1B	Hspa1b	NM_010478	-2.25 **
heat shock protein 105	Hsp105	NM_013559	-2.25 **
tubulin, beta 1, 2810484G07Rik	Tubb1		-2.15 *
Adipsin	Adn	NM_013459	-1.92 **
carbonic anhydrase 3	Car3	NM_007606	-1.91 **
DnaJ (Hsp40) homolog, subfamily B, member 1	Dnajb1	NM_018808	-1.90 **
ERBB receptor feedback inhibitor 1	Errfi1	NM_133753	-1.81 **
RIKEN full-length enriched library, clone:F830002E14 product: hypothetical Phenylalanine-rich region profile containing protein		AK089567	-1.69 **
fatty acid synthase	Fasn	NM_007988	-1.65 **
dickkopf homolog 3 (Xenopus laevis)	Dkk3	NM_015814	-1.61 *
Wnt inhibitory factor 1	Wif1	NM_011915	-1.60 **
glycoprotein 5 (platelet)	Gp5	NM_008148	-1.57 *
heat shock protein 1, alpha	Hspca	NM_010480	-1.53 **
	mt-Nd5		-1.52 *
adipocyte, C1Q and collagen domain containing	Acdc	NM_009605	-1.50 **
3-hydroxybutyrate dehydrogenase (heart, mitochondrial)	Bdh	NM_175177	-1.50 *
heat shock protein 1, beta	Hspcb	NM_008302	-1.49 **
4-aminobutyrate aminotransferase	Abat	NM_172961	-1.49 **
DNA-damage-inducible transcript 4	Ddit4	NM_029083	-1.49 **
cyclin-dependent kinase inhibitor 1A	Cdkn1a	NM_007669	-1.49 **
heat shock protein 1	Hspb1	NM_013560	-1.48 **
potassium voltage-gated channel, shaker-related subfamily, member 5	Kcna5	NM_145983	-1.46 **
CD9 antigen	Cd9	NM_007657	-1.45 *
protein phosphatase 1, regulatory (inhibitor) subunit 3C	Ppp1r3c	NM_016854	-1.44 **
RIKEN full-length enriched library, clone:2510042H12 product: weakly similar to RAT HEMOGLOBIN ALPHA CHAIN (FRAGMENT) [Rattus norvegicus]		AK011092	-1.44 *
immunoglobulin superfamily, member 1	Igsf1	NM_183336	-1.43 **
SRY-box containing gene 18	Sox18	NM_009236	-1.42 *
phosphatidylinositol (4,5) bisphosphate 5-phosphatase, A	Pib5pa	NM_172439	-1.41 *
transferrin receptor	Tfrc	NM_011638	-1.41 **
cysteine and histidine-rich domain (CHORD)-containing, zinc-binding protein 1	Chordc1	NM_025844	-1.40 **
eukaryotic translation elongation factor 2	Eef2	NM_007907	-1.40 **
FERM domain containing 5	Frmd5	NM_172673	-1.40 **
inhibitor of DNA binding 1	Idb1	NM_010495	-1.40 **
procollagen-proline, 2-oxoglutarate 4-dioxygenase (proline 4-hydroxylase), alpha 1 polypeptide	P4ha1	NM_011030	-1.40 **
protein O-fucosyltransferase 2	Pofut2	NM_030262	-1.40 *
1500015O10Rik		NM_024283	-1.40 **

*Data obtained with 2 high-iron and 2 control samples.

** Data obtained with 3 high-iron and 3 control samples.

From the lists of microarray results we selected 15 genes which presented the highest fold change values. The expression levels of these genes were then analyzed in the same mouse strain (C57BL/6) by Q-RT-PCR (quantitative reverse-transcription PCR). Certainly, the results from these analyses showed a good correlation between the two methods; the expression of all the genes was regulated and displayed the same direction of change. The fold change values obtained from PCR experiments were all over 1.4 except for the *Tfrc *gene whose downregulation in the skeletal muscle reached the value of -1.36.

**Figure 2:**
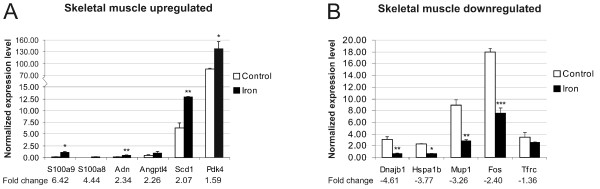
**Confirmation of microarray results for skeletal muscle by Q-RT-PCR**. The experiments were performed on samples derived from C57BL/6 male mice. The result values are expressed as mean of triplicate runs +/- standard deviation. Statistical significant differences relative to control diet fed mice were determined. *p < 0,05; **p < 0,01; ***p < 0,001. A, Q-RT-PCR analysis of 6 genes with up-regulated expression after iron overload. B, Q-RT-PCR evaluation of 5 genes with iron-induced down-regulation of expression by microarray analysis.

**Figure 3:**
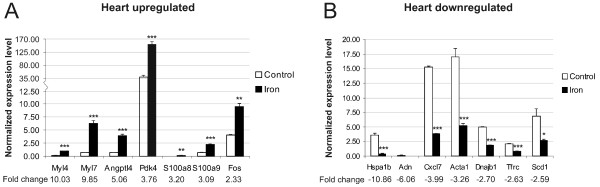
**Verification of data obtained for heart samples by microarray analysis using Q-RT-PCR**. Samples from C57BL/6 male mice were used in these experiments. The result values are expressed as mean of triplicate runs +/- standard deviation. Statistical significant differences relative to control diet fed mice were determined. *p < 0,05; **p < 0,01; ***p < 0,001. A, Q-RT-PCR evaluation of seven genes with up-regulated expression after iron overload. B, Q-RT-PCR analysis of seven genes with iron-induced down-regulation of expression by microarray.

Representations of these Q-RT-PCR results are depicted in figures 2 and 3. In general, the fold-change values obtained by microarray analysis were smaller than those determined by Q-RT-PCR. This phenomenon has been described previously and is probably due to the fact that array analyses are less quantitative than Q-PCR [21].

**Figure 4:**
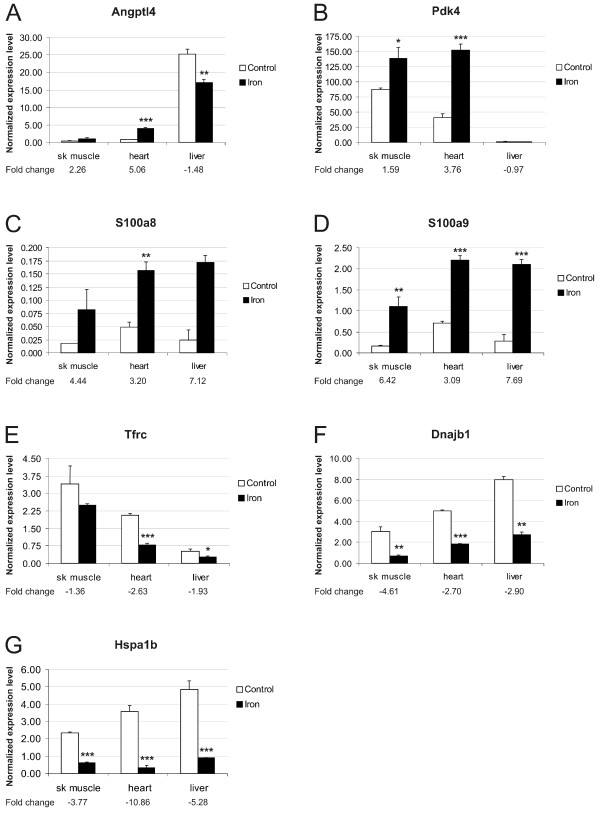
**Expression analysis of genes presenting same trend in muscular tissues and comparison with hepatic expression**. C57BL/6 male mice were used in this analysis. The result values are expressed as mean of triplicate runs +/- standard deviation. Statistical significant differences relative to control diet fed mice were determined. *p < 0,05; **p < 0,01; ***p < 0,001. A-D, Genes with up-regulated expression in both skeletal muscle and heart after iron overload. E-G, Genes with down-regulated expression in skeletal muscle and heart after iron overload.

The hepatic mRNA levels of these 15 genes were also analyzed by Q-RT-PCR. The results for genes whose expression varied in the same direction in both skeletal muscle and heart after iron loading are shown together with their expression in the liver in Figure 4. The expression of four of the 15 genes (*Myl4, Myl7, Acta1 and Adn*) was considered negligible in the liver because of very low signal intensity. Among the 11 remaining genes only the hepatic expression of *Pdk4 *(shown in figure 4B) and Cxcl7 (fold change of +1.38, data not shown) was not significantly regulated by dietary iron.

### Expression of genes involved in hepcidin regulatory pathway during dietary iron overload

One of the aims of this study was to explore the effect of dietary iron overload on the expression of the iron-related genes hemojuvelin (*Hjv*) and neogenin (*Neo*) in skeletal muscle and heart. We did not observe differential expression of these genes or any of the traditional iron-regulatory genes (such as *Cybrd1*, *Slc11a2*, *Slc40a1*, *Heph*, *Trfr2*, *Hfe *or *Hamp*) by the microarray approach, except for the transferrin receptor gene (*Tfrc*), which was down-regulated by iron in heart, skeletal muscle and liver (Figure 4E). Even though the microarray method we used proved to be very accurate, we wanted to verify these results and to explore the response of hepcidin expression in the studied tissues by Q-RT-PCR.

**Figure 5:**
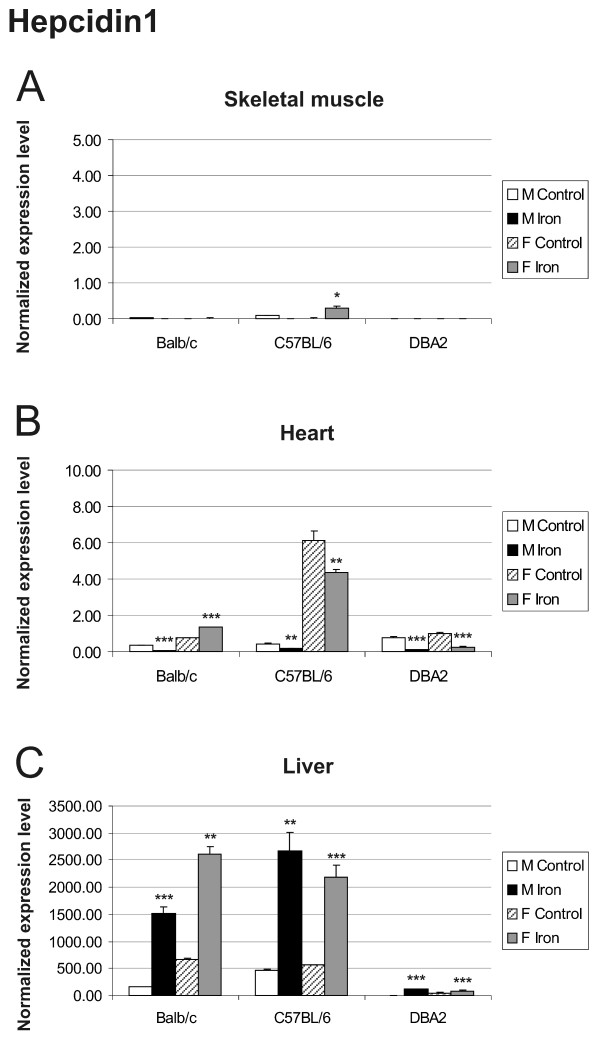
**Expression of hepcidin1 in skeletal muscle (A), heart (B) and liver (C) assessed by Q-RT-PCR**. The expression of hepcidin1 transcripts was assessed in control *versus *iron fed mice of 3 strains (Balb/c, C57BL/6, DBA2). The result values are expressed as mean of triplicate runs +/- standard deviation. Statistical significant differences relative to control diet fed mice were determined. *p < 0,05; **p < 0,01; ***p < 0,001. F = female; M = male.

**Figure 6:**
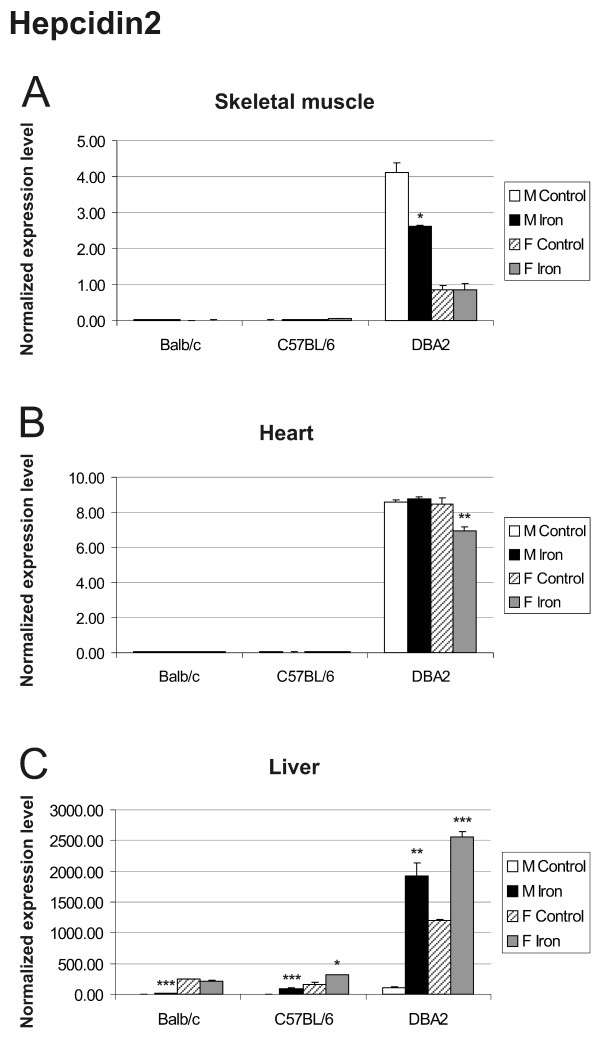
**Q-RT-PCR analysis of hepcidin2 mRNA expression in skeletal muscle (A), heart (B) and liver (C)**. The expression of hepcidin2 in control *versus *iron overloaded mice was analyzed in 3 strains (Balb/c, C57BL/6, DBA2). The result values are expressed as mean of triplicate runs +/- standard deviation. Statistical significant differences relative to control diet fed mice were determined. *p < 0,05; **p < 0,01; ***p < 0,001. F = female; M = male.

The expression of hepcidin1 and hepcidin2 in the liver was greatly up-regulated by iron overload and varied according to mouse strain and gender [22,23]. In Balb/c and C57BL/6 mice, hepcidin 1 was the predominant form expressed in the liver, while in DBA2 mice, the hepatic expression of hepcidin 2 was dominant (Figures 5C and Figure 6C). The expression of hepcidin1 in the skeletal muscle was negligible in all strains (Figure 5A). In the heart muscle, it showed a slight tendency towards decreased expression in most iron fed mice, although the baseline signal in control mice was already quite low (Figure 5B). Only DBA2 mice expressed hepcidin2 in the skeletal muscle and heart, and this expression was not clearly regulated by iron overload (Figure 6A and 6B).

**Figure 7:**
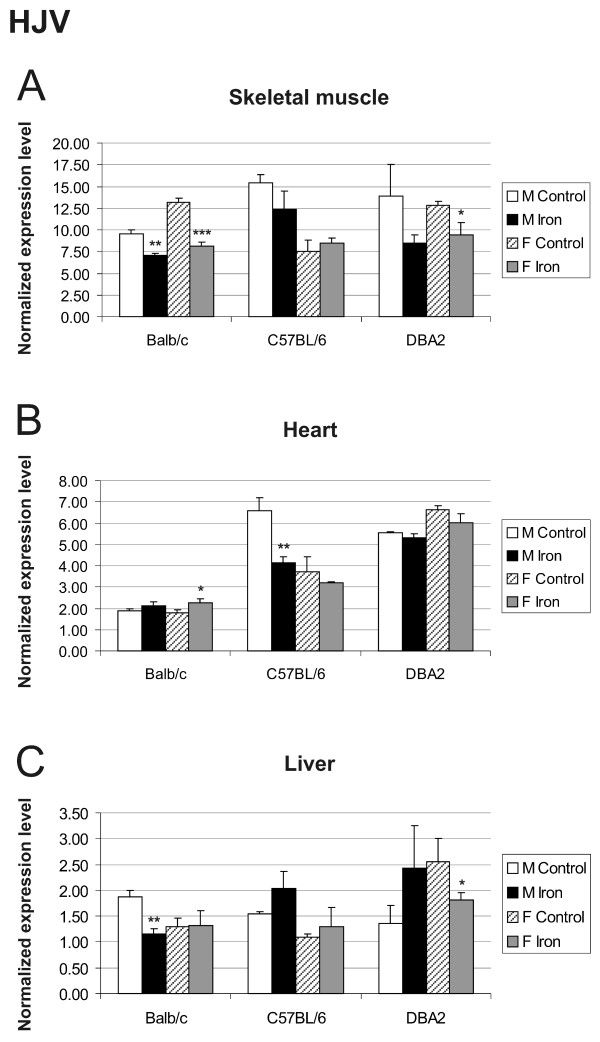
**Expression of hemojuvelin (HJV) in skeletal muscle (A), heart (B) and liver (C)**. Q-RT-PCR analysis of HJV mRNA levels in control *versus *iron overloaded mice of 3 strains (Balb/c, C57BL/6, DBA2). The result values are expressed as mean of triplicate runs +/- standard deviation. Statistical significant differences relative to control diet fed mice were determined. *p < 0,05; **p < 0,01; ***p < 0,001. F = female; M = male.

**Figure 8:**
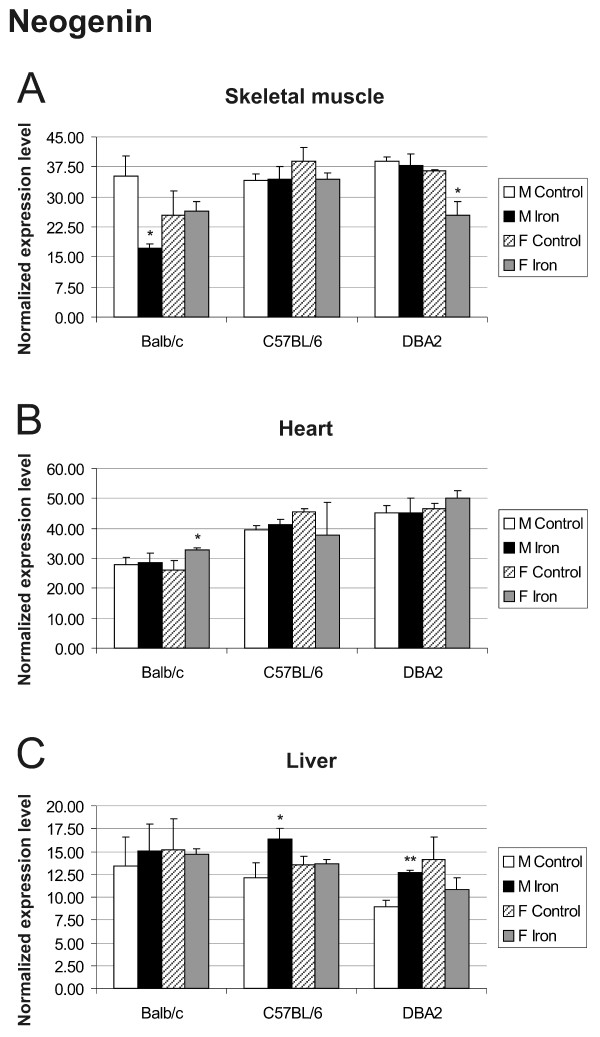
**Neogenin transcript levels in skeletal muscle (A), heart (B) and liver (C)**. 3 mouse strains (Balb/c, C57BL/6, DBA2) were used for this Q-RT-PCR analysis. The result values are expressed as mean of triplicate runs +/- standard deviation. Statistical significant differences relative to control diet fed mice were determined. *p < 0,05; **p < 0,01; ***p < 0,001. F = female; M = male.

The results for hemojuvelin expression did not indicate any clear regulation by iron overload, strain or gender in any of the tissues studied (Figure 7). This is in agreement with previous studies of hepatic expression [24,25]. Hemojuvelin expression only showed a minor trend downwards in skeletal muscle and heart of mice fed with high-iron diet. No significant changes were observed for neogenin expression (Figure 8).

## Discussion

Excess free iron participates in the formation of free radicals causing oxidative stress and cell damage, which is evidenced as a series of pathological manifestations [26]. While some studies have analyzed the effects of iron on the transcriptional profiles in liver and duodenum, this is the first study reporting changes in mRNA expression that may contribute to iron-induced effects on skeletal muscle and heart. We used a genome-wide mRNA expression profiling approach and validated the most substantive changes by Q-RT-PCR.

Expression of antioxidant enzymes is considered a protective mechanism against oxidative stress-induced damage. However, the regulation of antioxidant enzymes in response to oxidative stress is a rather controversial issue, as the results vary greatly depending on the type and length of the stimulus and the type of cells or tissue tested. We did not find increased expression of oxidative stress-related genes or antioxidant enzymes after iron overload, except for glutathione peroxidase 3, whose expression was induced by 1.35-fold change in heart (data not shown).

The data analysis identified two genes encoding calcium- and zinc-binding proteins, *S100a8 *(calgranulin A) and *S100a9 *(calgranulin B) among the up-regulated genes presenting the highest fold changes. These proteins form a rather ubiquitous heterodimer called calprotectin. The highest amounts of this protein complex are located in neutrophil granulocytes, monocytes and keratinocytes [27]. Calprotectin is a pro-inflammatory cytokine that is upregulated in many inflammatory conditions, and is involved in innate immunity, leukocyte adhesion, endothelial transmigration and processes of chronic inflammation [28]. *In vitro *studies have shown that reactive oxygen species (ROS) induce protein levels of S100a9 [29]. Additionally, S100a8 expression is induced in keratinocytes upon exposure to ultraviolet A (UVA) radiation, a stimulus causing oxidative stress. Interestingly, the antioxidant enzymes superoxide dismutase and catalase, whose mRNA expression was unaffected in our microarray, abrogate S100a8 induction [30]. We found that both S100a8 and S100a9 transcripts were substantially up-regulated in skeletal muscle, heart and liver of iron-loaded mice, although the levels of S100a8 transcript in skeletal muscle were negligible and, in general, both genes were weakly expressed. This demonstrates the high sensitivity and accuracy of both the microarray analysis and the Q-RT-PCR method. It is noteworthy that S100a8 and S100a9 transcripts showed a very similar up-regulation pattern in the tissues studied, which agrees with the concept of the two proteins forming a heterodimer. The transcriptional regulation of the *S100a8 *and *S100a9 *genes seems to be rather complex, with promoter binding sites for transcription factors such as activator protein 1 (AP-1), nuclear factor kappa B (NF-κB) and C/EBP. Consistently, at least AP-1 and NF-κB have been previously identified to be regulated by the intracellular redox state [31].

Pyruvate dehydrogenase kinase 4 (Pdk4) phosphorylates and inactivates the pyruvate dehydrogenase complex, decreasing the rate of glucose oxidation and thus increasing blood glucose levels. Increased Pdk4 expression and activity has been observed in both skeletal muscle [32,33] and heart [34] of insulin-resistant mouse models. The question arises of whether Pdk4 overexpression causes insulin resistance or *vice versa*. Insulin suppresses Pdk4 expression in skeletal muscle [35] and, according to a recent study by Kim and coworkers [36], this effect is impaired in insulin resistance, suggesting that insulin resistance may indeed induce Pdk4 expression. However, several studies using high-fat dietary models of insulin resistance indicate that Pdk4 overexpression occurs before the development of insulin resistance [37-39]. Although it has not been documented directly that increased Pdk4 mRNA levels can indeed cause insulin resistance, it seems possible that a vicious cycle may exist between these two phenomena. In the present study, we show an up-regulation of Pdk4 mRNA levels in skeletal muscle and heart but not in the liver of iron-loaded mice. Diabetes mellitus is the major endocrine disorder associated with HH. The mechanisms responsible for this clinical manifestation are still obscure, but two processes have been proposed: the pancreatic β-cell iron accumulation results in cell damage and diminished insulin secretion, and liver iron overload leads to insulin resistance [40]. The herein reported induction of Pdk4 expression in the skeletal and heart muscle might represent a novel mechanism involved in the development of diabetes mellitus in HH.

Angiopoietin-like 4 (Angptl4) is a secreted protein produced mainly in fat tissue, and to a lesser extent in liver, placenta, skeletal muscle and heart. It is directly involved in regulating glucose homeostasis, lipid metabolism, and insulin sensitivity. Angptl4 decreases the activity of lipoprotein lipase (LPL), thus inhibiting lipoprotein metabolism and increasing plasma triglyceride levels. Transgenic mice with Angptl4 overexpression directed to heart muscle (lipoprotein-derived fatty acids are the major energy source in this tissue) show reduced cardiac LPL activity, decreased triglyceride utilization and impaired cardiac function resulting in cardiomyopathy [41]. Transgenic overexpression of Angptl4 from a liver-specific promoter causes hypertriglyceridemia similar to that induced by adenoviral over-expression [42]. These results support the hypothesis that Angptl4 has LPL-dependent actions [43]. Accordingly, in LPL-expressing tissues (muscle, heart and adipose tissue) Angptl4 may bind directly and inactivate LPL, restricting acquisition of free fatty acids to these sites: it is not shed into plasma from these tissues but rather acts in an autocrine/paracrine fashion. On the other hand, in the liver, which has low LPL expression, Angptl4 is shed to plasma and inhibits LPL in other locations, causing a general reduction of triglyceride utilization and acting as an endocrine factor. Interestingly, we showed a 5-fold increase in the level of Angptl4 transcript in the heart of iron-loaded mice, raising the possibility that early induction of Angptl4 expression could contribute to the pathogenesis of cardiomyopathy in HH. The increased expression of Pdk4 and Angptl4 observed in skeletal muscle and heart of iron-loaded mice might have its origin in a common mechanism. The forkhead transcription factor Foxo1 is a major regulator of insulin action in insulin-sensitive tissues (liver, skeletal muscle and adipose tissues) and it is involved in insulin's action to suppress Pdk4 and Angptl4 [36,44].

Myosin light polypeptide 4 (Myl4) (encoding the alkali atrial essential light chain (ELCa)) and myosin light polypeptide 7 (Myl7) (encoding the regulatory light chain (RLC-A)) show a 10-fold up-regulation in the cardiac muscle of iron-loaded mice. Both genes belong to the EF-hand family of Ca^2+ ^binding proteins and are part of the myosin molecular complex. They appear to be involved in force development during muscle contraction. ELC is important in the interaction between myosin and actin [45]. There are two forms of ELC in the cardiac muscle, ELCa and ELCv (encoded by Myl3). ELCa has a higher performance than ELCv and its elevated accumulation in diseased heart is considered a compensatory response in heart failure [46]. Furthermore, transgenic rats overexpressing ELCa in the heart show an improvement in contractile parameters [47]. These observations open the possibility that the induction of cardiac Myl4 and Myl7 expression observed in our experiments is a compensatory response to early damage produced by iron accumulation. Additionally, according to our microarray results, other myosin genes were induced by iron in mouse heart (*Myh7*) and skeletal muscle (*Myl2*). Actin filaments play an essential role, along with myosin, in muscle contraction. Curiously, in the present work, iron suppressed the expression of skeletal muscle and smooth muscle isoforms of actin (acta1 and acta2) in the heart, as well as the cardiac isoform (actc1) in skeletal muscle.

The present microarray data analysis identified one gene (Stearoyl-coenzyme A desaturase 1, *Scd1*), which showed marked upregulation (1.75 fold) in the skeletal muscle and downregulation (-2.40 fold) in the heart after iron overload. This finding was also confirmed by Q-RT-PCR. Scd1 is an iron-containing enzyme with a central lipogenic role. It catalyzes the insertion of a double bond into fatty acyl-CoA substrates, the preferred one being stearoyl-CoA, and yielding oleoyl-CoA. Oleic acid is the major monounsaturated fatty acid of membrane phospholipids, triglycerides, cholesterol esters, wax esters and alkyl-1,2-diacylglicerol. The (stearic acid/oleic acid) ratio has important effects on cell membrane fluidity and signal transduction. The overexpression of Scd1 has been shown to be associated with genetic predisposition to hepatocarcinogenesis [48]. Scd1 mRNA levels were induced 2.49 times in mouse liver during iron overload (data not shown), an effect that was previously shown in both enteral and parenteral models of iron overload [49]. As Pigeon and coworkers have discussed, most likely the effect of iron on Scd1 expression in the liver is not direct, but a compensatory mechanism in response to the need to renew unsaturated fatty acids.

The FBJ osteosarcoma oncogene (Fos) is a major component of activator-protein-1 (AP-1), a redox-sensitive transcription factor complex, which also includes members of the Jun (c-Jun, JunB, JunD), Maf and ATF subfamilies. Fos is thought to have an important role in signal transduction, cell proliferation and differentiation. Expression of c-fos and c-jun can be induced by many stimuli and compounds, including some metals such as iron [50]. Accordingly, the present work shows increased expression of c-Fos in the heart and liver of iron-loaded mice. However, in skeletal muscle, c-Fos was down-regulated and, according to the microarray results, the same is true for c-Jun. Probably other mechanisms are influencing the transcription of c-Fos and c-Jun in skeletal muscle. Interestingly, a recent study suggested that c-Jun and JunB negatively regulate the transcription of *S100a8 *and *S100a9 *[51]. Furthermore, AP-1 activity had been previously connected to iron metabolism in several ways. For example, AP-1 regulates transcription of ceruloplasmin (the plasma iron oxidase) [52], and the promoter region of *HFE *contains an AP-1 transcription element [53].

Heat shock proteins, or stress proteins, are expressed in response to heat shock and a variety of other stress stimuli including oxidative free radicals and toxic metal ions. The members of the 70-kDa heat shock protein family (Hsp70) assist cells in maintaining functional proteins under stressful conditions [54]. Hsp40 proteins stimulate the ATPase activity of Hsp70 proteins and stabilize the interaction of these chaperons with their substrate proteins [55]. In the present study, dietary iron overload decreased the expression of *Hspa1b *(a member of Hsp70 family) and *Dnajb1 *(a member of Hsp40 family) in skeletal muscle, heart and liver of mice as validated by Q-RT-PCR. In accordance with these findings, our microarray results also showed decreased expression of several other heat shock protein genes in skeletal muscle (*Hsp105, Hspca, Hspb1 *and *Hspa1a*) and heart (*Hsp105, Hspcb, Hspca *and *Hspb1*) of iron-loaded mice. This unexpected result might represent novel regulatory mechanisms specific to these concrete experimental conditions.

The post-transcriptional regulation of transferrin receptor 1 and divalent metal transporter 1 by iron is mediated through iron-responsive elements located in the 3'-untranslated region of their mRNAs [56,57]. As expected, we found decreased Tfrc mRNA expression in skeletal muscle, heart and liver of iron-loaded mice but, surprisingly, the expression of divalent metal transporter 1 was not changed substantially.

The expression levels of hepcidin1 and hepcidin2 transcripts in the liver are markedly influenced by strain and gender, in accordance with previous reports [22,23]. DBA2 mice differ markedly in the expression levels of their hepcidin genes when compared with Balb/c and C57BL/6 mice. For DBA2, the difference in hepcidin2 expression was evident not only in liver, but also in the heart and skeletal muscle. These results further demonstrate that iron responses can vary between different mouse strains.

## Conclusion

To conclude, we have identified genes whose expression is altered in skeletal muscle and heart during iron overload. The number of the affected genes and the magnitude of the changes were relatively low, which is probably due to the fact that skeletal muscle and heart are not the primary targets of iron loading. Interestingly, some of the regulated genes identified in this study are involved in modulation of glucose and lipid metabolism, transcription and cellular stress responses. These might represent novel links between iron overload and the pathogenesis of cardiomyopathy and diabetes in HH. Further investigation of these genes may help to understand how iron excess leads to these common HH manifestations.

## Methods

### Animal care and experimental iron overload

The experiments with mice were performed in the laboratory animal centre of the University of Oulu. The mice were kept under specific pathogen-free conditions and the experiments were approved by the Animal Care and Use Committee of the University of Oulu (permission No 102/05). Five male and five female mice from each of three strains (Balb/c, C57BL/6, and DBA/2) were placed on a diet (Lactamin, Stockholm, Sweden) supplemented with 2% carbonyl iron (Sigma-Aldrich Sweden AB, Stockholm, Sweden, #C3518) at the age of 10–12 weeks. Equivalent groups of littermates were fed control chow diet without iron supplementation (0.02% iron). After 6 weeks of treatment, blood was collected from the mice under anaesthesia. Animals were then sacrificed and liver, skeletal muscle (extensor digitorum longus, EDL) and heart samples were immediately collected and immersed in RNAlater (Ambion, Huntingdon, UK). EDL is relatively easy to identify and isolate and it has been used as a reference muscle in many physiological studies. Liver samples were also collected and stored frozen before measurement of iron content.

### Determination of hepatic and cardiac iron content

Liver and heart tissue samples were analyzed for non-heme iron content using the bathophenanthroline method as described by Torrance and Bothwell [58]. The values are expressed as μg of iron per g dry weight.

### RNA isolation

Total RNA was obtained using RNeasy RNA isolation kit (Qiagen, Valencia, CA) as recommended by the manufacturer. Residual DNA was removed from the samples using RNase-free DNase (Qiagen). RNA concentration and purity were determined using optical density (OD) measurements at 260 and 280 nm. All the samples had an OD260/OD280 ratio of 1.95 or higher.

### Microarray analysis

Microarray studies were performed in the Finnish DNA Microarray Centre at Turku Centre for Biotechnology. Heart and skeletal muscle specimens derived from 3 male C57BL/6 mice of each group (iron diet and control diet) were subjected to total RNA extraction. The resulting samples were analyzed individually. 200 ng of total RNA from each sample was amplified using the Illumina™ RNA TotalPrep Amplification kit (Ambion) following the manufacturer's instructions. The *in vitro *transcription reaction, which was conducted for 14 h, included labelling of the cRNA by biotinylation.

### Hybridization and scanning

Labelled and amplified material (1.5 μg/array) was hybridized to Illumina's Sentrix Mouse-6 Expression BeadChips™ (Illumina, Inc., San Diego, CA) (12 samples, 2 chips) at 55°C for 18 h according to Illumina BeadStation 500X™ protocol. Arrays were washed and then stained with 1 μg/ml cyanine3-streptavidin (Amersham Biosciences, Buckinghamshire, UK). The Illumina BeadArray™ reader was used to scan the arrays according to the manufacturer's instructions. Samples were analyzed using the BeadStudio™ software from Illumina. The hybridization control report showed problems in 2 of the arrays, corresponding to 2 heart samples, one from a control mouse and the other from an iron-loaded mouse. In both cases, 228 probes failed to hybridize, and therefore, these probes were excluded from the analyses of these 2 samples.

### Data analysis

Array data were normalized with Inforsense KDE version 2.0.4 (Inforsense, London, UK) using quantile normalization method. The fold-change values were calculated for each gene using the same software. The resulting data were filtered according to a fold-change of 1.4 and -1.4 for up- and down-regulated expression, respectively. This value has been proposed as an adequate compromise above which there is a high correlation between microarray and quantitative PCR data, regardless of other factors such as spot intensity and cycle threshold [59].

### Quantitative real-time PCR

The RNA extracts from 5 mice within each study group were equally pooled and RNA samples (3 μg from liver and 1.5 μg from heart and skeletal muscle) were converted into first strand cDNA with a First Strand cDNA Synthesis kit (Fermentas, Burlington, Canada) using random hexamer primers according to the protocol recommended by the manufacturer. The relative expression levels of target genes in mouse liver, skeletal muscle and heart were assessed by quantitative real-time RT-PCR using the Lightcycler detection system (Roche, Rotkreuz, Switzerland). The validations of microarray data were performed on samples obtained from C57BL/6 mice, while mRNA expression of hepcidin1, hepcidin2, hemojuvelin and neogenin was studied in three strains (Balb/c, C57BL/6, and DBA/2). Four housekeeping genes (*Actb *(β-actin), *Gapdh *(glyceraldehyde-3-phosphate dehydrogenase), *Hprt1 *(hypoxanthine phosphoribosyl-transferase I), and *Sdha *(succinate dehydrogenase complex subunit A)) were used as internal controls to normalize the cDNA samples for potential quality and quantity differences. The primers for the housekeeping genes and for mouse *Hjv *and *Neo *target genes have been described earlier [19]. Mouse Hamp1 and Hamp2 primers have been also previously characterized [60]. The primer sets for the remaining target genes in this study are shown in Table 5. Most of them were designed using Primer3 [61], based on the complete cDNA sequences deposited in GenBank. The specificity of the primers was verified using NCBI Basic Local Alignment and Search Tool (Blast) [62]. When possible, and in order to avoid amplification of contaminating genomic DNA, both primers from each set were specific to different exons.

**Table 5:** Sequences of the primers used in this study

**Symbol**	**Name**	**GenBank Accession No**.	**Forward primer (5'-3')**	**Reverse primer (5'-3')**	**Source**
*Angptl4*	Angiopoietin – like 4	NM_020581	CACGCACCTAGACAATGGA	AGAGGCTGGATCTGGAAA	*
*Pdk4*	Pyruvate dehydrogenase kinase, isoenzyme 4	NM_013743	GATTGACATCCTGCCTGACC	TCTGGTCTTCTGGGCTCTTC	*
*S100a8*	Calgranulin A, S100 calcium binding protein A8	NM_013650	GGAAATCACCATGCCCTCTAC	GCCACACCCACTTTTATCACC	*
*S100a9*	Calgranulin B, S100 calcium binding protein A9	NM_009114	CGACACCTTCCATCAATACTC	GAGGGCTTCATTTCTCTTCTC	*
*Fos*	FBJ osteosarcoma oncogene	NM_010234	CGGGTTTCAACGCCGACTA	TTGGCACTAGAGACGGACAGA	RTprimerDB, 3328
*Myl4*	Myosin light polypeptide 4	NM_010858	GGGTAAAGCACGTTTCTCCA	AGGGAAGGTTGTGGGTCAG	*
*Myl7*	Myosin light polypeptide 7	NM_022879	TCACCGTCTTCCTCACACTC	GCTGCTTGAACTCTTCCTTG	*
*Acta1*	Actin alpha 1	NM_009606	CCAAAGCTAACCGGGAGAA	CCCCAGAATCCAACACGA	*
*Cxcl7*	Chemokine (C-X-C motif) ligand 7	NM_023785	GCCCACTTCATAACCTCCA	ATCACTTCCACATCAGCACA	*
*Tfrc*	Transferrin receptor 1	NM_011638	TCATGAGGGAAATCAATGATCGTA	GCCCCAGAAGATATGTCGGAA	QPPD, 1607
*Scd1*	Stearoyl-Coenzyme A desaturase 1	NM_009127	TGGGTTGGCTGCTTGTG	GCGTGGGCAGGATGAAG	QPPD, 1847
*Adn*	Adipsin, complement factor D	NM_013459	AACCGGACAACCTGCAATC	CCCACGTAACCACACCTTC	*
*Mup1*	Major urinary protein 1	NM_031188	CTCTATGGCCGAGAACCAGA	AGCGATTGGCATTGGATAGG	*
*Dnajb1*	DnaJ (Hsp40) homolog, subfamily B, member 1	NM_018808	CGACCGCTATGGAGAGGAA	GCCACCGAAGAACTCAGCA	*
*Hspa1b*	Heat shock protein 1B	NM_010478	GAGGAGTTCAAGAGGAAGCA	GCGTGATGGATGTGTAGAAG	*

* designed using Primer3

Each PCR reaction was performed in a total volume of 20 μl containing 0.5 μl of first strand cDNA, 1× of QuantiTect SYBR Green PCR Master Mix (Qiagen, Hilden, Germany), and 0.5 μM of each primer. Amplification and detection were carried out as follows: After an initial 15-min activation step at 95°C, amplification was performed in a 3-step cycling procedure: denaturation at 95°C, 15 s, ramp rate 20°C/s; annealing temperature determined according to the melting temperature for each primer pair, 20 s, ramp rate 20°C/s; and elongation at 72°C, 15s, ramp rate 20°C/s for 45 cycles and final cooling step. Melting curve analysis was always performed after the amplification to check PCR specificity. To quantify the levels of transcripts in the studied tissues, a standard curve was established for each gene using 5-fold serial dilutions of known concentrations of purified PCR products generated from the same primer sets. Every cDNA sample was tested in triplicate and the obtained crossing point (Cp) value facilitated the determination of the levels of starting message using a specific standard curve. The geometric mean of the 4 internal control genes was used as an accurate normalization factor for gene expression levels [63]. The normalization factor is always considered as a value of 100 and the final result is expressed as relative mRNA expression level.

### Statistical analyses

The mean values and standard deviations were calculated from the individuals in each group for the iron measurements and from technical triplicates for the Q-RT-PCR experiments. The Student's *t*-test (unpaired, 2-tailed) was used to analyze statistically the differences in iron content and in gene expression between control and iron loaded mice. Theoretically, the Q-PCR technology used herein can detect a minimum of 100 copies of starting material. In order to avoid wrong use of the statistical methods, these were not applied to data with raw values below 300 copies.

## List of Abbreviations

BMP- bone morphogenetic protein;

ELC- essential light chain;

HH- hereditary hemochromatosis;

LPL- lipoprotein lipase;

OD- optical density;

Q-RT-PCR - quantitative reverse-transcription PCR;

RGM- repulsive guidance molecule;

RLC- regulatory light chain.

## Authors' contributions

AR participated in sample collection and preparation, designed primers, carried out the Q-RT-PCR and drafted the manuscript. MH performed microarray data analysis. LK carried out microarray data analysis. REF, RSB and BRB provided materials, participated in experimental design and made critical reviewing of the manuscript. SP conceived the study, participated in its design and coordination, participated in sample collection and made critical reviewing of the manuscript. All authors read and approved the final manuscript.
